# Smartphone-Based Digital Peer Support for a Walking Intervention Among Public Officers in Kanagawa Prefecture: Single-Arm Pre- and Postintervention Evaluation

**DOI:** 10.2196/53759

**Published:** 2024-09-24

**Authors:** Masumi Okamoto, Yoshinobu Saito, Sho Nakamura, Makoto Nagasawa, Megumi Shibuya, Go Nagasaka, Hiroto Narimatsu

**Affiliations:** 1 Graduate School of Health Innovation Kanagawa University of Human Services Kawasaki Japan; 2 Center for Innovation Policy Kanagawa University of Human Services Kawasaki Japan; 3 Cancer Prevention and Control Division Kanagawa Cancer Center Research Institute Yokohama Japan; 4 Faculty of Sport Management Nippon Sport Science University Yokohama Japan; 5 Graduate School of Physical Education, Health and Sport Studies Nippon Sport Science University Tokyo Japan; 6 Department of Genetic Medicine Kanagawa Cancer Center Yokohama Japan; 7 A 10 Lab Inc Tokyo Japan

**Keywords:** digital health, mhealth, ehealth, smartphone app, smartphone application, peer support, digital peer support, social support, group intervention, physical activity, health promotion, behavior change, apps, step counting, workplace health

## Abstract

**Background:**

Digital peer support, defined as peer support delivered through technology such as smartphone apps, may be promising to promote activity in the form of step counts. Interactions among users have a positive impact on retention rates, and apps with social elements show significant improvements in daily step count. However, the feasibility of digital peer support in promoting physical activity (PA) is unknown; therefore, its effectiveness on step count and the clinical implications remain unconfirmed.

**Objective:**

This study aimed to assess the feasibility of digital peer support over a 3-month intervention period using the retention rate as the outcome. Moreover, changes in daily step count and physical measurements were compared between pre- and postintervention.

**Methods:**

The study design was a 3-month 1-arm intervention with participants from local government offices in Kanagawa, Japan. We used an available smartphone app, Minchalle, as the tool for the group intervention. Participants were required to report their daily step count to a maximum of 5 members composed exclusively of study participants. The primary outcome was the retention rate. Secondary outcomes included daily step count, the rate of achieving daily step goals, physical measurements, and lifestyle characteristics. Descriptive statistics and the Pearson coefficient were used to examine the relationship between goal achievement and step count, as well as changes in step count and various variables including physical measurements.

**Results:**

Of the 63 participants, 62 completed the intervention. The retention rate was 98% (62/63). The average daily step count during the intervention was 6993 (SD 2328) steps, an 1182-step increase compared with the count observed 1 week before the intervention began. The rate of achieving the daily step count during the intervention was 53.5% (SD 26.2%). There was a significant correlation (*r*=0.27, *P*=.05) between achieving daily step goals and increasing daily step count. Comparative analyses showed that changes in weight (68.56, SD 16.97 kg vs 67.30, SD 16.86 kg; *P*<.001), BMI (24.82, SD 4.80 kg/m^2^ vs 24.35, SD 4.73 kg/m^2^; *P*<.001), somatic fat rate (28.50%, SD 7.44% vs 26.58%, SD 7.90%; *P*=.005), systolic blood pressure (130.42, SD 17.92 mm Hg vs 122.00, SD 15.06 mm Hg; *P*<.001), and diastolic blood pressure (83.24, SD 13.27 mm Hg vs 77.92, SD 11.71 mm Hg; *P*=.002) were significantly different before and after the intervention. Similarly, the daily amount of PA significantly improved from 5.77 (SD 3.81) metabolic equivalent (MET)–hours per day to 9.85 (SD 7.84) MET-hours per day (*P*<.001).

**Conclusions:**

This study demonstrated that digital peer support is feasible for maintaining a high retention rate and can, therefore, effectively promote PA. It can be a promising tool to improve daily step count, subjective PA, and clinical outcomes, such as weight and somatic fat rate.

**Trial Registration:**

UMIN Clinical Trials Registry UMIN000042520; https://tinyurl.com/46c4nm8z

## Introduction

Interventions for health promotion and behavioral changes using smartphone apps may be more cost-effective than face-to-face services due to their affordability and accessibility. The usefulness of smartphone apps for increasing physical activity (PA), including step count, is evident. Previous studies have reported that smartphone app interventions had a positive impact on both objective and subjective PA [1-4], including walking time [5,6].

Peer support refers to the process through which individuals who share common experiences or face similar challenges come together as equals to offer and receive help based on the knowledge that comes through shared experiences [7]. This includes psychological support such as acceptance and encouragement. To date, face-to-face peer support has been effective at increasing PA, including step count, in the healthy general population [8]. Conversely, peer support also poses challenges for in-person interventions owing to the high travel costs and required organization and human resources.

In contrast, smartphone apps are inexpensive and accessible, making it possible to provide services to a multitude of people at a lower cost than face-to-face meetings. To overcome the challenges of conventional peer support, digital peer support—defined as peer support delivered through technology and media such as smartphone apps [9]—was recently developed. Previous studies have indicated that communication with peers and social support [10] may have a positive impact on increasing step count [11]. Therefore, digital peer support may be promising to promote PA in the form of step counts. 

To date, the feasibility of or engagement with digital peer support is unclear, although the use of smartphones is related to the magnitude of their effects [4,12]. Most previous studies defined engagement as the retention rate, by calculating the percentage of users who continued using smartphone apps during the intervention [13]. However, there is no consensus on measuring engagement; attempts have been made for conceptualization [14-16]. In general, the retention rates with health care smartphone apps for PA are low, at approximately 45% [17], and the use of smartphone apps has declined over time [1,18].

Social features of smartphone apps, such as social support and social comparison through interactions among users, reportedly have a positive impact on engagement, and they are considered potential leverage to increase the retention rate [18-20]. However, no studies have verified the retention rate of specialized digital peer support; therefore, its effectiveness on step count remains unconfirmed. In a single-arm, pre-post comparison study that examined the efficacy of smartphone apps, in which social networking was the main component, the retention rate among 55 participants was 82% during a 6-month intervention [21]. Another 2 intervention arms (gamification and basic apps) in a 100-day, 3-group randomized control trial (RCT) evaluated the efficacy of a smartphone app equipped with gamification, including social interaction among users, on promoting PA [12]. In that study, attrition (30 days of nonuse) was reported for approximately 32% and 39% of the gamified and basic app groups, respectively, meaning the retention rates were 68% and 61%, respectively [16]. Moreover, the gamified app group demonstrated significantly increased subjective PA at the 9-month follow-up compared with the control group who engaged in individual walking. However, the effect on objective step count was not revealed [22].

We aimed to assess the effectiveness of digital peer support as a tool for promoting health. This study evaluated the feasibility of digital peer support over a 3-month intervention period using retention rate as the outcome. We also measured its effectiveness. Specifically, changes in physical measurements, PA, lifestyle characteristics, and psychosocial factors were compared between pre- and postintervention. Additionally, previous studies revealed that goal setting and self-monitoring contributed to increased step count [4,23,24]. Hence, this study also examined the relationship between the goal attainment rate and increasing step count using digital peer support. If its effectiveness was demonstrated, it would help individuals increase their step count easily, at a low cost, and remotely. Furthermore, digital peer support may be used as an intervention tool during medical consultation intervals for patients with lifestyle-related diseases or others for whom walking is effective for disease prevention or control.

## Methods

### Study Design and Procedures

This 3-month, 1-arm intervention study adopted a pre-post evaluation design. Baseline assessments, which consisted of paper-based questionnaires and physical measurements, were conducted in gymnasiums and other facilities arranged by the 7 municipalities in Kanagawa Prefecture and Kanagawa local government offices that participated in the study. The measurements were taken by the researchers or local municipal officials in Kanagawa Prefecture and Kanagawa local government officials trained by the researchers. Subsequently, the participants installed and set up a smartphone app called Minchalle (“Challenge yourself together with others” in Japanese) to commence the intervention.

Participants were required to send objective step count data for 1 week before the intervention as a baseline and during the 3-month intervention period via Minchalle. Objective step count data were measured using Google Fit (Google LLC) for Android users and Healthcare for iPhone users (Apple Inc), both capable of automatically counting steps while the phone is carried. Once Minchalle was connected to these apps, daily step count data were automatically transferred to and displayed in Minchalle. Following the 3-month intervention, participants were again required to complete paper-based questionnaires and undergo physical measurements as part of an endline survey. Endline measurements were performed in the same manner as the baseline assessments. In addition, to assess the validity of smartphone-measured data, step count measurements via an accelerometer (Active style Pro HJA-750C, OMRON Corporation) were conducted for 1 week before the intervention. Participants were required to wear the accelerometer around their waist while awake during this period. The study was conducted between December 2020 and June 2021.

### Recruitment and Participants

This study was announced through email or online in the offices of 7 local municipalities in Kanagawa Prefecture and Kanagawa local government offices in Japan. Interested participants participated in briefing sessions to deepen their understanding of this study, and, if eligible, paper-based informed consent was obtained. Inclusion criteria were participants aged between 20 years and 75 years who owned and were accustomed to using a smartphone and could communicate in Japanese. Exclusion criteria were participants who were currently pregnant, had physical or medical problems that did not allow safe participation in the walking intervention, had experience using Minchalle over the past 6 months, or had undergone another face-to-face or digital interventional study for PA promotion. The recruitment was conducted between December 2020 and April 2021.

### Intervention

Minchalle was commercially developed by a health care company (A 10 Lab) to help users foster desirable habits. It is available on both Android and iPhone. Its development was guided by the social cognitive theory based on the need to increase self-efficacy, a key driver of behavior adoption and maintenance. To enhance self-efficacy, Minchalle adopted a group intervention to maximize the mechanisms of social support and comparison among group members, which thereby increased the retention rate and made substantial achievements. Based on this basic concept, the main function of Minchalle was to facilitate group interactions, limiting each group to a maximum of 5 members and a minimum of 3 members with similar goals. All users were required to share their daily achievements with other members in a chat box, accompanied by a photo taken on the same day as evidence of their activity. Users could also enjoy interactions with other members by commenting or chatting at any time, although this was not obligatory. This platform was designed to help users continue desired behaviors by receiving praise and encouragement from other members for their achievements and gaining inspiration from other members’ activities. Apart from group interaction, the examples of functions installed in Minchalle were goal setting, self-monitoring, reminders through push notifications, rewards for achievements such as exclusive stickers available within this app, and coins that could be used for donations to organizations that implement projects with social significance. 

In this study, once the Minchalle app was installed, participants were anonymously assigned by researchers to a group that consisted of a maximum of 5 members. Each participant set their daily step goals based on their step counts from the past few days, without any instruction from the researchers. The intervention began with participants posting a photo of their total step count once a day in the group chat. To easily identify study participants, all the team members were composed exclusively of study participants. For their cooperation, participants were provided access to a premium version of Minchalle by A 10 Lab worth ¥500 (US $3.42) during the intervention period.

### Sample Size

In a similar pilot study in Australia measuring the effectiveness of a social networking mobile app on improving PA, there were 55 participants. Therefore, we set a target of approximately 50 participants.

### Measures

This study aimed to evaluate the feasibility of digital peer support interventions using retention rates. The secondary endpoints were the effectiveness of the intervention, measured via the change in step count between baseline and endline, and the association with the achievement rate of daily step goals.

#### Retention Rate

In this study, the retention rate was defined as the percentage of users who continued using the smartphone app during the 3-month intervention period. This indicator was used based on its common use in previous studies [13]. Owing to the setting up of Minchalle, participants were automatically removed from peer groups if they failed to report daily step counts or share any photos or comments for 8 consecutive days. Therefore, participants who were dismissed but returned to the team were counted as participants, and only participants who were dismissed and never returned were considered as having dropped out.

#### Daily Step Count

Objective daily step count data were obtained from Google Fit for Android users and HealthKit for iPhone users. All participants were required to submit their step data via Minchalle at both baseline and endline. The validity of Google Fit and Healthcare was confirmed, although interpretation should be done cautiously owing to the possibility of an underestimation of 10% to 20% due to noncarrying time [25-27]. Participants were asked to carry their smartphones for as long as possible while they were awake, live their daily lives, and report all the data at the end of the survey via the smartphone app. In this study, the positive impact on daily step count was examined by comparing the average daily step count during the week before and during the intervention. To calculate the average daily step count, the total daily step count was divided by the number of intervention days. Days with 0 steps were omitted to prevent underestimation, as there was a possibility that participants had forgotten to carry their smartphones that day. Moreover, the analysis included days with low step counts, as it was difficult to distinguish whether it reflected a step count issue or genuinely low PA.

#### Achievement Rate of Daily Step Goals

At the beginning of the intervention, participants were asked to set their daily step goals based on their step counts from the past few days, without any instructions from the researchers. These goals could be adjusted by the participants at any time during the study. The achievement rate was assessed by calculating the proportion of days on which participants met their daily step goal relative to the total number of intervention days.

#### Other Measurements

This study used a self-administered questionnaire to obtain sociodemographic characteristics, which included sleep, subjective PA, alcohol consumption, smoking, diet, personality and psychological traits, psychological stress, social relationships, quality of life, and physical checkups. These questionnaires were used in the Kanagawa ME-BYO prospective cohort study, a large population-based genomic cohort study to clarify gene–environmental interactions in noncommunicable diseases in collaboration with the Japan Multi-Institutional Collaborative Cohort (J-MICC) study [28]. Details of the ME-BYO cohort and J-MICC study and the relationship between the two have been described elsewhere [28-30]. Questionnaires on loneliness and self-efficacy were also used in this study.

Height and weight were measured to the nearest 0.1 cm and 0.1 kg, respectively, and BMI was calculated. Systolic blood pressure (SBP) and diastolic blood pressure (DBP) were measured via a sphygmomanometer. Lifestyle factors included subjective PA, smoking habits, alcohol consumption, and hours slept per day. We defined subjective PA as the sum of the daily amount of PA, estimated by multiplying the amount of time spent walking (3.0 metabolic equivalents [METs]) and engaging in hard labor (4.5 METs) by their assigned daily MET intensities, and 3 types of leisure-time activities (3.3 METs for light effort, 4.0 METs for moderate effort, and 8.0 METs for vigorous effort), calculated by multiplying the daily frequency, duration, and intensity. We also assessed the METs for engaging in hard labor (4.5 METs) alone. Detailed information on the calculation method has been provided in previous studies [31-33].

The Kessler Psychological Distress Scale is a 6-item screening scale with a 5-point rating that evaluates psychological distress in the preceding month. Higher scores indicate higher levels of psychological distress. Its validity and reliability have been described previously [34]. Social support items were derived from the ENRICHD Social Support Inventory (ESSI) [35]. The original version of the ESSI includes 7 items measured with a 5-point rating to assess the 4 domains of social support: emotion, instrument, information, and appraisal. Individual items are summed for a total score, with higher scores demonstrating greater social support. This study adopted a modified version of the ESSI that included 6 items to prevent duplication of the question on marital status. The Japanese version of the Short-Form Health-Related Quality of Life Health Survey was used to measure health-related quality of life. Details regarding this assessment have been previously described [36]. The Japanese version of the UCLA Loneliness Score was used, which consists of 20 items answered on a 4-point scale, with higher scores indicating a higher degree of loneliness [37,38]. Self-efficacy was assessed using the Generalized Self-Efficacy Scale [39], which comprises 23 items rated on a 5-point scale to measure belief in one’s competence to cope with a broad range of stressful or challenging demands [40]. Higher scores on this scale indicate a higher degree of self-efficacy.

### Statistical Analyses

Descriptive analyses were conducted to calculate the retention rate and rate of achieving daily step goals. The average daily step counts 1 week before and during the intervention period were calculated to measure the impact of the intervention, and the differences between the 2 were assessed. Pearson coefficients were used to assess the correlation between the rate of achieving daily step goals and the difference in average step counts before and during the intervention. Differences in weight, BMI, somatic fat rate, SBP, DBP, PA, lifestyle characteristics, and psychosocial factors between baseline and endline were analyzed via a 2-tailed *t* test. Furthermore, Pearson coefficients were examined between changes in average daily step counts and various variables, including physical measurements, lifestyle, and psychosocial factors, separately at baseline and endline. This enabled us to determine the variables to be used for subsequent multiregression analyses by omitting the possibility of multicollinearity among potential independent variables. Multiple regression analyses were conducted to demonstrate the impact of changes in daily step counts on health measurements, lifestyle, and psychosocial factors (Model 1), after adjusting for sex and age (Model 2), Model 2 + household income (Model 3), and Model 3 + average daily step count during the 1 week prior to the intervention (Model 4). Data analysis was performed using R software (version 4.2.2).

### Ethics Approval

This study was approved by the Institutional Review Board of Kanagawa University of Human Services (30-018). The study was registered in the UMIN Clinical Trials Registry (UMIN000042520). All participants provided written informed consent. Data were collected in a face-to-face setting, and all user data were deidentified before analysis.

## Results

### Participants’ Characteristics

**Figure 1:**
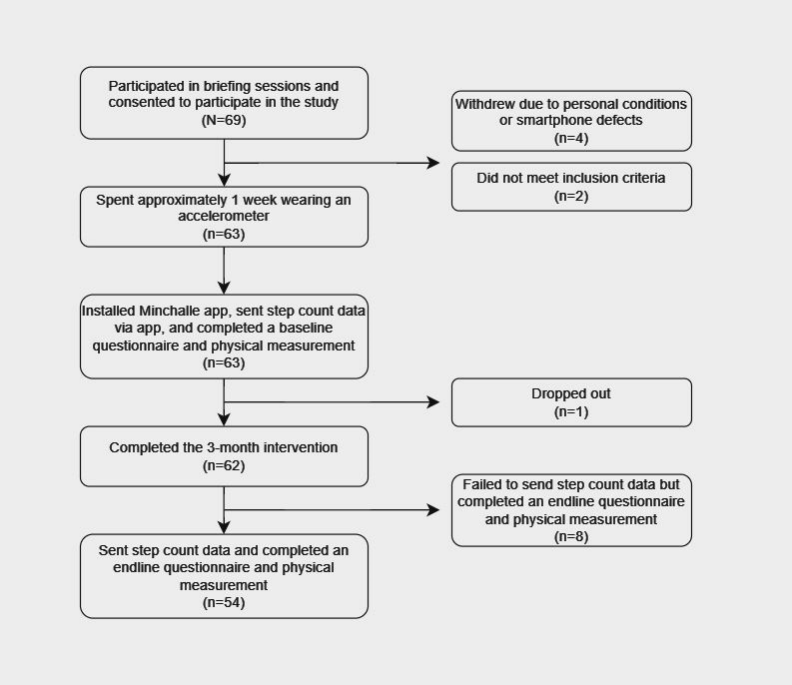
Study flowchart.

Figure 1 shows the study flowchart. Table 1 presents the baseline characteristics of the 62 participants. Participants’ ages ranged from 24 years to 63 years, with a mean age of 43.6 (SD 10.72) years. More than one-half (32/62, 52%) were men, and most (45/62, 73%) had a university degree or higher.

**Table 1:** Demographic characteristics of the 62 eligible participants.

Characteristics		Results, n (%)
**Age (years)**		
	20-29	8 (13)
	30-39	14 (23)
	40-49	18 (29)
	50-59	19 (31)
	60-69	3 (5)
**Sex**		
	Male	32 (52)
	Female	30 (48)
**Education**		
	High school	5 (8)
	Vocational school	5 (8)
	College	7 (11)
	University	38 (61)
	Graduate school	7 (11)
**Job category**		
	Administrator	42 (68)
	Manager	9 (15)
	Technical staff	10 (16)
	Other	1 (2)
**Marital status**		
	Married	47 (76)
	Single	14 (23)
	Others	1 (2)

### Retention Rate

Among the 69 participants who signed the consent forms, 4 withdrew shortly after the intervention began due to personal conditions or smartphone defects, 2 did not meet the eligibility criteria, and 1 dropped out in the middle. Therefore, 63 participants installed the app and started the intervention, and the retention rate was 98% (62/63).

Data on daily step count were received from 54 (87%) of the 62 participants at the 3-month endline, and 8 participants failed to share step count data at endline due to misunderstanding the procedures of the survey when completing the intervention and endline questionnaires. Thus, data from 54 participants were used for step count analysis.

### Improvement in Step Count

The average daily step counts during the week before the intervention began were 5811 (SD 2477) steps, as measured by Healthcare for iPhone or Google Fit by Android, and 7073 (SD 2301) steps, as measured by the accelerometer. Comparing the average daily step count from the smartphone app between baseline and during the intervention, an 1182-step increase was observed. The changes in average daily step count according to sex, age, and BMI at baseline are shown in Tables S1 and S2 in Multimedia Appendix 1.

### Achievement Rate of Daily Step Goals and Effect on Increase in Step Count

The mean daily step goal at baseline was 6808 (SD 1668) steps. The mean difference between the daily step goal and step count before the intervention began was 997 steps (*P*=.002). The rate of achieving the daily step count during the intervention was 53.5% (SD 26.2%). A significant correlation (*r*=0.27, *P*=.05) was observed between achieving daily step goals and an increase in daily step count. There was a statistically negative correlation between the difference in the daily step goal and pre-intervention step count and an increase in daily step count (*r*=–0.77, *P*<.001).

### Other Outcomes

Table 2 shows the comparative results of changes in health measurements, lifestyle characteristics, and psychosocial factors. Weight, BMI, somatic fat rate, SBP, and DBP were significantly different. Similarly, the daily amount of PA and engaging in hard labor significantly improved. However, no significant differences were observed in lifestyle characteristics nor psychosocial factors. The results of the groupwise analyses between those who maintained or increased their step count and those with a decreased step count during the intervention are summarized in Table S3 in Multimedia Appendix 1.

Table 3 shows the correlations between changes in step count, health measurements, and lifestyle characteristics measured at baseline and endline. A significant difference was observed between weight and BMI (*r*=–0.41, *P*=.002 for both variables). The correlations between the changes in step count and psychosocial factors are shown in Table S4 in Multimedia Appendix 1. Table 4 shows the results of the linear regression analysis. The increment in step count was significantly associated with weight, BMI, and SBP, even after adjusting for sex and age (Model 2: *P*=.02 for weight and BMI; *P*=.008 for SBP), household income (Model 3: *P*=.04 for weight; *P*=.03 for BMI; *P*=.02 for SBP), and all covariates (Model 4: *P*=.02 for weight and BMI; *P*=.01 for SBP). The results of the linear regression analysis to assess the association between changes in average daily step count and psychosocial factors are summarized in Table S5 in Multimedia Appendix 1.

**Table 2:** Differences in health measurements, lifestyle characteristics, and psychosocial factors between baseline and endline after the 3-month intervention (n=62).

Objective variables		Baseline, mean (SD)	Endline, mean (SD)	*P* value	
Weight (kg), mean (SD)		68.56 (16.97)	67.30 (16.86)	<.001	
BMI (kg/m^2^), mean (SD)		24.82 (4.80)	24.35 (4.73)	<.001	
Somatic fat rate (%), mean (SD)		28.50 (7.44)	26.58 (7.90)	.005	
SBP^a^ (mm Hg), mean (SD)		130.42 (17.92)	122.00 (15.06)	<.001	
DBP^b^ (mm Hg), mean (SD)		83.24 (13.27)	77.92 (11.71)	.002	
Daily PA^c^ (MET^d^-hours per day), mean (SD)		5.77 (3.81)	9.85 (7.84)	<.001	
Daily amount of engaging in hard labor (MET-hours per day), mean (SD)		1.31 (1.80)	3.59 (6.08)	.004	
**Alcohol consumption, n (%)**					.17^e^
	Yes	33 (53)	24 (39)		
	No	29 (47)	37 (61)		
Length of sleep (hours), mean (SD)		6.04 (0.95)	6.09 (0.97)	.95	
ESSI^f^, mean (SD)		23.66 (5.37)	22.84 (5.65)	.27	
Physical function (Locomo 5), mean (SD)		0.90 (1.72)	0.75 (1.67)	.45	
Depression and anxiety (K6^g^), mean (SD)		4.52 (5.00)	4.58 (5.34)	.89	
**Psychological stress, n (%)**					.46^h^
	High	22 (35)	19 (32)		
	Moderate	35 (57)	31 (52)		
	Low	5 (8)	9 (15)		
	Not at all	0	1 (1)		
Loneliness, mean (SD)		41.97 (8.92)	42.61 (10.46)	.41	
Self-efficacy, mean (SD)		69.98 (11.47)	70.56 (13.33)	.38	
Health related QOL^i^ (EQ-5S), mean (SD)		0.91 (0.10)	0.89 (0.15)	.33	

^a^SBP: systolic blood pressure.

^b^DBP: diastolic blood pressure.

^c^PA: physical activity.

^d^MET: metabolic equivalent.

^e^Chi-squared analysis (n=61 at endline).

^f^ESSI: ENRICHD Social Support Instrument.

^g^K6: Kessler Psychological Distress Scale.

^h^Trend test (n=60 at endline).

^i^QOL: quality of life.

**Table 3:** Pearson coefficients for the relationships between the change in average daily step count and the differences in physical measurements and lifestyle characteristics between baseline and endline (n=54).

Objective variables^a^	Correlation coefficient	*P* value
Weight (kg)	–0.41	.002
BMI (kg/m^2^)	–0.41	.002
Somatic fat rate (%)^b^	–0.15	.31
SBP^c^ (mm Hg)^d^	–0.26	.06
DBP^e^ (mm Hg)^d^	–0.02	.90
Daily PA^f^ (MET^g^-hours per day)	–0.11	.45
Daily amount of engaging in hard labor (MET-hours per day)	–0.15	.27
Alcohol consumption^d^	N/A^h,i^	.13
Length of sleep (hours)^b^	0.14	.31

^a^The exact intervention period varied from participant to participant: n=11: 90 days; n=10: 92 days; n=5: 93 days; n=13: 94 days; n=6: 98 days; n=2: 99 days; n=7: 101 days.

^b^n=51.

^c^SBP: systolic blood pressure.

^d^n=52.

^e^DBP: diastolic blood pressure.

^f^PA: physical activity.

^g^MET: metabolic equivalents.

^h^N/A: not applicable.

^i^Jonckheere-Terpstrata trend test.

**Table 4:** Linear regression analyses of the effect of the differences of physical measurements and lifestyle characteristics on the average daily step count at baseline and endline after the 3-month intervention.

Objective and explanatory variables			Model 1^a^ (n=53)						Model 2^b^ (n=53)							Model 3^c^ (n=46)							Model 4^d^ (n=46)			
			Regression coefficient^e^		*P* value			Regression coefficient^e^			*P* value			Regression coefficient^e^				*P* value			Regression coefficient^e^				*P* value	
**Weight (kg)**						.002						.02							.04							.02
	Change in step count	–0.4006		N/A^f^			–0.4152			.002			–0.3399				.02			–0.4639				.004		
	Sex	—^g^		—			–0.1370			.81			–0.5151				.38			–0.7247				.22		
	Age	—		—			0.0176			.51			–0.0146				.65			–0.0061				.85		
	Household income	—		—			—			—			0.0014				.12			0.0014				.12		
	Step count at baseline	—		—			—			—			—				—			–0.0002				.09		
**BMI (kg/m^2^)**						.002						.02							.03							.02
	Change in step count	–0.1467		N/A			–0.1510			.002			–0.1253				.02			–0.1695				.005		
	Sex	—		—			–0.1294			.53			–0.2662				.23			–0.3409				.13		
	Age	—		—			0.0062			.53			–0.0061				.62			–0.0030				.80		
	Household income	—		—			—			—			0.0005				.10			0.0005				.11		
	Step count at baseline	—		—			—			—			—				—			–0.0001				.11		
**Somatic fat rate (%)^h^**						.31						.45							.32							.35
	Change in step count	–0.2511		N/A			–0.2006			.42			0.0015				.99			–0.1399				.66		
	Sex	—		—			–0.1791			.87			–1.2000				.32			–1.4732				.24		
	Age	—		—			–0.0639			.22			–0.1247				.07			–0.1135				.10		
	Household income	—		—			—						0.0013				.48			0.0012				.50		
	Step count at baseline	—		—			—			—			—				—			–0.0003				.33		
**SBP^i^ (mm Hg)^j^**						.06						.008							.02							.01
	Change in step count	–1.9820		N/A			–1.8190			.07			–2.0350				.09			–2.9610				.03		
	Sex	—		—			11.7100			.01			13.7358				.01			11.9093				.02		
	Age	—		—			–0.3203			.13			–0.0904				.75			–0.0361				.90		
	Household income	—		—			—			—			–0.0904				.12			–0.0122				.12		
	Step count at baseline	—		—			—			—			—				—			–0.0018				.13		
**DBP^k^ (mm Hg)^j^**						.91						.33							.42							.57
	Change in step count	–0.1019		N/A			–0.2195			.79			–0.3749				.68			–0.2971				.78		
	Sex	—		—			6.7060			.07			7.6480				.06			7.8010				.07		
	Age	—		—			0.0461			.79			0.0471				.83			0.0426				.85		
	Household income	—		—			—			—			0.0008				.89			0.0008				.90		
	Step count at baseline	—		—			—			—			—				—			0.0002				.87		
**Daily PA^l^ (MET^m^-hours per day)**						.45						.09							.22							.21
	Change in step count	–0.3979		N/A			–0.1993			.69			–0.3899				.51			–0.0133				.98		
	Sex	—		—			–4.1360			.07			–3.1282				.22			–2.4910				.34		
	Age	—		—			–0.1678			.12			–0.0524				.71			–0.0784				.58		
	Household income	—		—			N/A			—			–0.0049				.21			–0.0048				.22		
	Step count at baseline	—		—			—			—			—				—			0.0007				.24		
**Daily amount of engaging in hard labor (MET-hours per day)**						.28						.02							.10							.17
	Change in step count	–0.4305		N/A			–0.2576			.49			–0.2591				.56			–0.1970				.70		
	Sex	—		—			–3.6871			.03			–3.3468				.08			–3.2419				.10		
	Age	—		—			–0.1460			.07			–0.0953				.36			–0.0996				.35		
	Household income	—		—			—			—			–0.0028				.34			–0.0027				.35		
	Step count at baseline	—		—			—			—			—				—			0.0001				.80		
**Alcohol consumption^n^**						.30						.23							.36							.44
	Change in step count	–0.0694		N/A			–0.0621			.35			–0.0001				.40			–0.0851				.28		
	Sex	—		—			0.1476			.43			0.1471				.45			0.1162				.56		
	Age	—		—			–0.0139			.12			–0.0153				.14			–0.1394				.19		
	Household income	—		—			—			—			0.0001				.78			0.0001				.74		
	Step count at baseline	—		—			—			—			—				—			<–0.0001				.47		
**Length of sleep (hours)^h^**						.31						.30							.17							.22
	Change in step count	0.0557		N/A			0.0460			.41			0.1246				.04			0.0995				.15		
	Sex	—		—			0.2990			.12			0.0906				.65			0.0658				.75		
	Age	—		—			–0.0053			.57			–0.0117				.29			–0.0104				.35		
	Household income	—		—			—			—			–0.0001				.73			–0.0001				.75		
	Step count at baseline	—		—			—			—			—				—			<–0.0001				.43		

^a^Simple regression analysis.

^b^Multiregression analyses adjusted for sex and age.

^c^Multiregression analyses adjusted for sex, age, and household income.

^d^Multiregression analyses adjusted for all covariates (sex, age, household income, and step count at baseline).

^e^Number of 1000-step changes in each objective variable.

^f^N/A: not applicable.

^g^Not included in the model.

^h^Model 1: n=51; model 2: n=51; model 3: n=44; model 4: n=44.

^i^SBP: systolic blood pressure.

^j^Model 1: n=52; model 2: n=52; model 3: n=45; model 4: n=45.

^k^DBP: diastolic blood pressure.

^l^PA: physical activity.

^m^MET: metabolic equivalent.

^n^Dichotomous variable: drinking alcohol at both baseline and endline or drinking only at baseline but not at endline.

## Discussion

### Principal Findings

This study demonstrated the feasibility, as indicated by the retention rate, of digital peer support. The finding that 98% of the participants continued to use the smartphone app even after the 3-month intervention shows that the retention rate in this study was higher than that of any other smartphone app equipped with the function of group interaction for promoting PA. A meta-analysis reported that engagement was generally low, while the retention rate of online social networks varies depending on the number of participants and the duration of the intervention [41]. A systematic review that examined the impact of social networks reported retention rates ranging from 60% to 80% [42]. An RCT assessing the efficacy of a smartphone app that facilitates interaction among users regarding walking over a 100-day intervention period reported that retention rates in the 60th percentile were higher than those in previous studies [16]. In a similar study evaluating a single-arm, 6-month intervention targeted at university students, in which user interaction was one of the components, the retention rate was 82% [21]. Considering these figures, digital peer support was effective at maintaining a high retention rate and can, therefore, be a promising tool for affordable and accessible health promotion. A reason may be that daily interaction, including social support and competition among participants and mutual comparison, serves as a positive factor for the prolonged use of a smartphone app, as shown in previous studies [18]. Another reason may be that the intervention period of this study was relatively short. Previous research has pointed out that the retention rate of smartphone apps generally remains high in short-term interventions that last from a few weeks to several months but tends to decrease after approximately 6 months from the beginning of the intervention [3]. We have conducted an RCT with a 6-month intervention period (UMIN000046936) to assess the effectiveness of digital peer support, including its long-term effects.

This study also showed that digital peer support had the potential to increase PA. Participants' daily average step count (+1182 steps), subjective daily amount of PA (+4.08 METs), and daily amount of engagement in hard labor (+2.28 METs) increased during the intervention period compared with 1 week before the intervention. Although step count was lower than those in a meta-analysis [4], which showed that an average 13-week intervention increased the number of steps per day by 1850, these results were consistent with those of other studies that showed increased objective PA [3,6]. The high retention rate in this study may have led to a significant increase in the amount of PA. These results indicate that digital peer support improved PA and daily step count. Additionally, this study demonstrated that the percentage of goal attainment was 53.5% (SD 26.2%). Furthermore, the statistical correlation between the rate of achieving daily step goals and the amount of increase in daily step counts was significant (*r*=0.27, *P*=.05), which was consistent with a previous study that reported that self-regulating, such as goal setting and self-monitoring, was a factor that influenced an increase in step count. Apart from self-regulation, previous studies revealed that other behavior change techniques, such as feedback, were also effective at improving PA [43]. Therefore, future studies should examine the impact of other behavior change techniques on digital peer support.

This study also suggested that digital peer support had the potential to improve health outcomes. Pre- and postintervention comparisons showed reduced weight (–1.26 kg), BMI (–0.47 kg/m^2^), somatic fat rate (–1.92%), SBP (–8.42 mm Hg), and DBP (–5.32 mm Hg). These results were consistent with meta-analyses that examined the effectiveness on health outcomes of smartphone apps that engage individuals [44]. Weight, BMI, and SBP could have decreased owing to the increased step count that resulted from smartphone app use. Participants’ mean age in this study was 43.8 years, which was similar to that in a previous study that found significant weight loss in participants with a mean age of 45 years or older [6]. However, it remains unclear in which age group digital peer support is particularly effective for promoting health outcomes. Changes in health outcomes are currently being investigated in our ongoing RCT (UMIN000046936), which will also examine which demographic backgrounds are likely to contribute to improved health outcomes.

### Limitations and Future Direction

This study demonstrated the possibility that digital peer support played an important role in increasing step count and thereby improved health outcomes with a high retention rate. However, several challenges remain unaddressed. First, it was a single-arm intervention. Since this study used a before-and-after comparison, other factors that may have affected step count and health outcomes could not be excluded. Furthermore, the effectiveness could not be verified in detail. Therefore, we are currently conducting an RCT (UMIN000046936). Second, the daily step count measured by smartphone apps may be underestimated compared with their true value. Our findings revealed a difference of approximately 18% in daily step count during the week before the intervention between the smartphone app (5811 steps) and accelerometer (7073 steps). This finding was consistent with those of previous studies that found that the number of steps measured by smartphones was 12% to 20% lower than the true value depending on where on the body the phone was being carried or due to intermittent bouts of activity [25,26]. Therefore, our daily step count results during the intervention should be interpreted based on these limitations. Third, a previous study reported that the retention rate for apps with social features is related to personal preferences. For example, users’ intent to use smartphone apps and a positive attitude toward technology, which are influenced by the simplicity or user-friendliness of the apps, were identified as positive factors for engagement [45,46]. The stage of behavioral change also affected the continued use of apps [18]. As this study did not collect information on personal preferences, future research should examine the effects of personal traits on the continued use of digital peer support. Fourth, the study did not elucidate participants’ specific backgrounds and lifestyles, examples of which were age, BMI [16], and being less physically active [21]. Although there was a slight increase in daily step count among middle-aged or older individuals or those who were overweight before the intervention, these were not statistically significant. However, future research is required to verify the groups or demographics more likely to increase their step count using digital peer support. Fifth, the intervention was conducted between the winter and spring seasons, and the impact of seasonality on PA was not considered, while previous research demonstrated PA level variations across the seasons, with a higher PA level in the summer compared with other seasons [47]. The possibility of seasonal and environmental effects should be considered when designing interventions in future research. Finally, the sample size in this study was 69 participants, which might have been underpowered to measure changes in health outcomes. The sample size was not calculated in this study because there were no prior studies examining the effectiveness of digital peer support on health outcomes. Future studies are needed to assess the effectiveness of digital peer support on both step count and health outcomes, so that effect sizes can be calculated appropriately.

### Conclusion

This study demonstrates that digital peer support is feasible for maintaining high retention rates and can, therefore, effectively promote PA. Digital peer support can be a promising tool from a clinical perspective for improving daily step count, subjective PA, and health outcomes, such as weight, BMI, somatic fat rate, and blood pressure.

## Appendix

Multimedia Appendix 1 Supplementary tables.

## Abbreviations

DBP: diastolic blood pressure
ESSI: ENRICHD Social Support Inventory
J-MICC: Japan Multi-Institutional Collaborative Cohort
METs: metabolic equivalents
PA: physical activity
RCT: randomized controlled trial
SBP: systolic blood pressure
