# Supplementary material for: Smartphone-Based Digital Peer Support for a Walking Intervention Among Public Officers in Kanagawa Prefecture: Single-Arm Pre- and Postintervention Evaluation
Source: JMIR Form Res. 2024 Sep 24;8:e53759. doi: 10.2196/53759 (PMC11462101; doi:10.2196/53759)
Supplement: Supplementary file 1 [file formative_v8i1e53759_app1.docx]

Supplemental table 1. The transition of average daily step count by sex and age category between baseline and during the intervention period

	Male					Female				
Age category	N	Baseline*		Intervention period		N	Baseline*		Intervention period	
		Mean	SD	Mean	SD		Mean	SD	Mean	SD
20-29	3	8,982	NA	8,837	NA	5	4,313	1,348	5,472	1,909
30-39	9	5,462	2,119	6,492	1,735	5	5,098	2,199	6,575	2,186
40-49	10	6,761	2,188	6,839	1,083	8	6,119	1,432	7,345	2,262
50-59	9	6,979	5,236	9,224	4,611	10	5,885	2,333	6,695	1,834
60-69	1	4,197	NA	5,775	NA	2	3,837	2,280	7,626	213

* Figure at baseline shows the average daily step counts during 1 week before the intervention.

Supplementary table 2.The transition of average daily step count by BMI category between baseline and during the intervention period

BMI category	Male						Female					
		Baseline*			Intervention period			Baseline*			Intervention period	
	N	Mean	SD	N	Mean	SD	N	Mean	SD	N	Mean	SD
Underweight(<18.5)	0	N/A**	N/A**	0	N/A**	N/A**	2	4,866	929	3	5,668	528
Healthy Weight (18.5-24.9)	18	6,451	1,991	18	6,778	1,679	20	5,604	2,161	19	6,947	2,231
Overweight (25-)	14	6,158	3,778	14	7,916	3,453	8	5,055	1,688	8	6,398	1,473

* Figures at baseline show the daily average step counts during 1 week before the intervention.

** N/A: not applicable

Supplemental table 3. The difference in health measurements, lifestyle characteristics, and psychosocial factors at baseline and endline; group-wise analyses between those with step count being maintained or increased and those with decreased during the intervention to the level of baseline

Objective variables		Step count maitained or increased (N=37)		Step count decreased (N=16)		p value
		mean	SD	mean	SD	
Weight (kg)		-1.67	2.23	-0.33	1.63	<.001
BMI (kg/m2)		-0.63	0.81	-0.12	0.63	<.001
somatic fat rate(%)*		-2.54	3.91	-1.63	3.47	<.001
sBP (mmHg)**		-11.81	16.71	-4.50	17.31	<.001
dBP (mmHg)**		-6.33	14.11	-4.81	10.93	<.001
Daily amount of PA (METs-hour per day)		3.26	6.2	5.48	11.9	<.001
Daily amont of engaging in hard labor (METs-hour per day)		1.22	3.81	4.64	9.6	.04
Alcohol comsumption#						
	Yes (n)	12		6		1.00
	No(n)	24		10		
Sleeping hour*		0.04	0.62	-0.14	0.78	<.001
ESSI		-1.69	5.64	-0.12	5.88	.04
Physical function (Locomo 5)		-0.03	1.76	-0.38	1.86	<.001
Depression and anxiety (K6)**		-0.53	4.22	0.93	3.59	.13
Psychological Stress**#	High(n)	13		6		.60
	Moderate(n)	15		8		
	Low(n)	7		1		
	Not at all(n)	1		0		
Lonliness		0.43	5.18	1.62	7.45	.95
Self efficacy**		0.25	7.45	0.81	4.82	.96
Health related QOL (EQ-5S)*		-0.03	0.16	-0.02	0.1	<.001

*Number of participants with step count maintained or increased was 35

**Number of participants with step count maintained or increased was 36 and those with step count decreased was 15

#Figures in the row of mean showed the cases of each category and the column of p-value was the result of χ^2 analyses

Supplemental table 4. Pearson's coefficients between change in daily average step count and difference in psychosocial factors between baseline and endline

Objective variables	Correlation coefficient	p value
ESSI	-0.18	.20
Physical function (Locomo 5)	-0.10	.47
Depression and anxiety (K6)	-0.20	.15
Psychological Stress	N/A*	.81
Lonliness	0.13	.35
Self efficacy	0.07	.60
Health related QOL (EQ-5S)	0.14	.34

* Trend test (jonckheere.test) was conducted and N/A indicated not applicable

Supplemental table 5. Linear regression of differences in psychosocial factors on difference in daily average step count at baseline and endline after 3-month intervention

Objective and explanatory variables***	Model 1 *		Model 2**		Model 3**		Model 4**	
	Regression coefficient$	p value	Regression coefficient$	p value	Regression coefficient$	p value	Regression coefficient $	p value
**ESSI**		0.20		0.35		0.84		0.93
Change in step count	-0.4571		-0.5442	0.14	-0.3286	0.42	-0.3255	0.49
Sex			-0.1775	0.91	-0.7175	0.69	-0.7124	0.07
Age			0.0978	0.20	0.0716	0.40	0.0714	0.41
Household income					-0.3291	0.91	0.0714	0.91
Step count at baseline							0.0714	0.99
**Physical function (Locomo 5)**	-0.0817	0.47		0.91		0.97		0.92
Change in step count			-0.0792	0.50	-0.0801	0.57	-0.1498	0.35
Sex			-0.0707	0.89	-0.0001	0.82	-0.2513	0.69
Age			-0.0019	0.94	-0.0001	0.89	0.0004	0.99
Household income					-0.0401	0.97	0.0208	0.98
Step count at baseline							0.0208	0.36
**Depression and anxiety (K6)**	-0.4577	0.17		0.09		0.14		0.22
Change in step count			-0.3506	0.28	-0.1599	0.69	-0.2307	0.61
Sex			-2.1761	0.06	-2.8552	0.04	-2.9086	0.04
Age			-0.0460	0.06	-0.0713	0.24	-0.0680	0.27
Household income					-0.0713	0.73	-0.0680	0.27
Step count at baseline							-0.0001	0.74
**Psychological Stress**	-0.0012	0.97		0.48		0.65		
Change in step count			0.0068	0.81	-0.0089	0.78	0.0084	0.81
Sex			-0.0020	0.99	0.0195	0.89	0.0275	0.85
Age			-0.0095	0.12	-0.0101	0.19	-0.0078	0.24
Household income					0.0002	0.36	-0.2895	0.19
Step count at baseline							<0.0001	0.31
**Loneliness**	0.3475	0.35		0.55		0.86		0.84
Change in step count			0.4242	0.27	0.0003	0.46	0.5039	0.30
Sex			-0.9717	0.56	-1.2755	0.49	-0.9622	0.61
Age			-0.0734	0.36	-0.0578	0.51	-0.0700	0.43
Household income					-1.7923	0.54	-1.9630	0.50
Step count at baseline							0.0004	0.40
**Self efficacy**	0.2683	0.60		0.96		0.78		0.82
Change in step count			0.2755	0.61	0.4965	0.44	0.7544	0.32
Sex			-0.3666	0.85	-1.4975	0.52	-1.3043	0.58
Age			0.0133	0.89	-0.0204	0.85	-0.0325	0.76
Household income					-4.1594	0.25	-4.4332	0.23
Step count at baseline							0.0004	0.50
**Health related QOL (EQ-5S)**	0.0092	0.34		0.74		0.88		0.92
Change in step count			0.0095	0.33	0.0107	0.34	0.0123	0.33
Sex			-0.0211	0.61	-0.0333	0.49	-0.0309	0.53
Age			-0.0006	0.77	-0.0010	0.65	-0.0012	0.63
Household income					-0.0111	0.88	-0.0124	0.87
Step count at baseline							-0.0124	0.78

*Model 1 showed simple regression analysis result.

**Model 2, 3, and 4 demonstrateed the results of multi-regression analyses adjusting for sex and age(Model2), sex, age and household income(Moderl3), and all covariates(sex, age, household income and step count at baseline)(Model4) respectively.

***Bold letter showed objective variables while normal font represents explanatory variables.
